# Limb heaviness as a sensorimotor disorder alters rehabilitation adherence after a stroke

**DOI:** 10.3389/fneur.2022.840808

**Published:** 2022-08-18

**Authors:** Yuanyuan Chen, Hongyan Yang, Yanqin Chen, Hui Wei, Meijuan Lan

**Affiliations:** Department of Nursing, The Second Affiliated Hospital of Zhejiang University School of Medicine, Hangzhou, China

**Keywords:** limb heaviness, rehabilitation adherence, sensorimotor disorder, stroke, nursing

## Abstract

**Introduction:**

To the best of our knowledge, it is still unknown how perceived limb heaviness affects rehabilitation adherence. As rehabilitation adherence is very important for the functional recovery of patients with stroke, it is important to explore the relationship between perceived limb heaviness and rehabilitation adherence.

**Methods:**

We retrospectively reviewed the data of patients with consecutive stroke recruited in the CIRCLE study. The influence of age, gender, time from onset to enrollment, educational background, hypertension, diabetes, Modified Rankin Scale (MRS), and National Institutes of Health Stroke Scale (NIHSS) on rehabilitation adherence was analyzed. Multiple linear regression analysis was used to examine the association between perceived limb heaviness and rehabilitation adherence changes.

**Results:**

A total of 108 participants completed the study. About 40 (37.0%) participants felt limb heaviness. The mean scores on the Medical Research Council (MRC) scale for the upper affected limb strength were 3.05 ± 1.7, and the mean score on the exercise adherence questionnaire (EAQ) was 34.27 ± 8.9. Univariate analysis showed that rehabilitation adherence levels differed in upper limb muscle strength and whether they perceived limb heaviness. After adjustment for independent predictors, we found that perceived limb heaviness was associated with rehabilitation adherence (*B* = −9.681 ± 1.494, *p* < 0.05) and *R*^2^ was 0.332 and 0.074 if the muscle strength of the upper limb and perceived limb heaviness were included in the model and the model was without perceived limb heaviness, respectively.

**Conclusion:**

By identifying patients with stroke with limb heaviness, it led to lower levels of motor functional rehabilitation adherence. We must pay more attention to limb heaviness and provide effective interventions to improve rehabilitation adherence and promote patient recovery.

## Introduction

Stroke is the leading cause of mortality and disability worldwide, with low- and middle-income countries (LMICs) accounting for 89% of all stroke-related disability-adjusted life-years (1). Many guidelines have recommended rehabilitation to improve functional recovery (2), which requires long-term rehabilitation (3). Rehabilitation often lasts more than 6 months and needs a lot of passion to persist. As rehabilitation plays an important role in the recovery of patients with stroke with functional disabilities, adherence becomes critical.

Adherence is defined as “the behavior of a patient in accordance with medical instructions” (4). Adherence is proposed to be the key link between an intervention and the achieved outcomes (5). Adherence to rehabilitation can significantly lower the risk of cardiovascular disease and mortality (6). Increased adherence to rehabilitation has been associated with improved functional recovery after a mild to moderate stroke (7). Many studies have demonstrated a positive dose–response relationship between adherence and functional outcomes after a stroke (5, 8, 9). All of these emphasize the importance of rehabilitation adherence after a stroke.

However, rehabilitation was often hampered by low adherence rates (10). As a result, it is very important to improve rehabilitation adherence. However, a study found that only 31% of patients with stroke actually performed the recommended exercises (11). A systematic review of 101 publications on web-based interventions in different areas (chronic conditions, lifestyle, and mental health) found an average adherence rate of 50% (12). As a result, the rate of rehabilitation adherence is low. To improve it, it is necessary to explore the factors that may affect rehabilitation adherence.

Adherence to rehabilitation in stroke is influenced not only by disease-specific factors, such as spasticity, fatigue, and paresis, but also by psychosocial factors (13–15). Perceived recovery, psychological aspects, support systems, professional supervision, and exercise prescription also influence adherence in stroke survivors (16, 17). Perceptual factors were the main factors that may affect rehabilitation adherence.

Limb heaviness is also one of the perceptual phenomena, where patients with stroke experience the affected limb as feeling heavier (18). Research by Kuppuswamy indicated that limb heaviness was a commonly perceptual phenomenon in patients with stroke (19). It was not yet known whether perceived limb heaviness as a perceptual factor affected rehabilitation adherence. Clarifying the relationship between them may find a new way to improve the level of rehabilitation adherence. Thus, we hypothesized that perceived limb heaviness would influence rehabilitation adherence. To the best of our knowledge, this is the first study to investigate the relationship between rehabilitation adherence and perceived limb heaviness. In this study, we aimed to screen patients with stroke for the presence of limb heaviness and to evaluate rehabilitation adherence by administering the exercise adherence questionnaire (EAQ) to patients with stroke. We also explored whether perceived limb heaviness alters rehabilitation adherence.

## Methods

### Sample and procedures

In this study, we retrospectively reviewed data from patients with consecutive stroke recruited into the CIRCLE study (20) (Clinical Trials. gov ID: NCT03702452) between 21 November 2018 and 19 November 2019. The CIRCLE study was to verify that nursing-directed rehabilitation in patients with stroke can compensate for the shortage of professional rehabilitation therapists (21). Inclusion criteria were: (1) patients who were between 18 and 90 years old; (2) patients who were diagnosed as having an ischemic stroke by computed tomography (CT) or magnetic resonance imaging (MRI) and who met the diagnostic criteria of the World Health Organization (WHO); (3) patients who had an initial ischemic stroke within 7 days, with limb dysfunction (muscular strength of the limbs is less than 5); (4) patients who maintain consciousness (the National Institutes of Health Stroke Scale (NIHSS) scale consciousness level 0 or 1); and (5) patients who signed an informed consent form. Exclusion criteria were: (1) patients whose blood vessel was recanalized after thrombolysis or thrombectomy; (2) patients who had a cardiopulmonary dysfunction; a history of craniocerebral trauma, fracture trauma, or rheumatoid arthritis; or already had a physical disability or other diseases that impacted the affected limb; and (3) patients who had a cognitive impairment or other mental illness that prevent cooperation with researchers. All patients with stroke underwent rehabilitation therapy after 24 h of hospital admission.

### Ethics statement

All subjects had provided written informed consent before participating in the study, and the protocols had been approved by the human ethics committee of The Second Affiliated Hospital of Zhejiang University. All clinical investigations were conducted in accordance with the principles outlined in the Declaration of Helsinki.

### Evaluation

All participants received the questionnaire on perceived limb heaviness and functional rehabilitation adherence. Demographic data (age, gender, time from onset to enrollment, educational background, hypertension, and diabetes) were collected from medical records. We also collected the Modified Rankin Scale (MRS) and the NIHSS.

Muscle strength of the affected limbs was assessed by the Medical Research Council (MRC) Scale. Biceps brachii and triceps brachii were accessed to represent the upper limb muscle and quadriceps femoris was assessed to represent the lower limb muscle. All patients with stroke had three attempts, and the best result was considered the outcome.

### Administration of the perceived limb heaviness

Perceived limb heaviness has two questions, asking participants to agree or disagree with the statement, “My limbs can become very heavy.” “My limbs can become very heavier than the other side” (19). Participants were instructed to answer the questions based on how they felt in the past 1 week. If participants felt that their limbs became very heavy or heavier than the other side, it was considered to be perceived limb heaviness. In this study, perceived limb heaviness was administered independently to all participants. All participants were divided into two groups based on whether they perceived limb heaviness.

### Administration of EAQ to patients with stroke

The EAQ was developed based on the exercise guidelines for patients with stroke in the USA, which is based on a large number of references (22), with a content validity index of 0.95 and an internal consistency coefficient of Cronbach's α 0.9 (23). It includes three major aspects, physical exercise adherence, exercise monitoring adherence, actively seeking advice, and 14 items, each item scored from 1 to 4, scores ranging from 14 to 56, the higher the score, the higher the adherence. The rate of adherence = (the actual score/theoretical high score × 100%) was divided into three levels: high adherence (>75%), moderate adherence (50–75%), and low adherence (<50%) (24). Patients with stroke are the primary patients who complete this questionnaire. Only when they cannot complete it themselves, family members complete it for them. Participants were instructed to answer the questions based on how they felt in the past 1 week.

### Statistical analysis

Data analysis was conducted using SPSS 24.0. All statistical tests were two-tailed, and an alpha of 0.05 was taken to indicate significance. Descriptive statistics were used to report the demographic and clinical characteristics of patients with stroke, including age, gender, time from onset to enrollment, educational background, hypertension, diabetes, limb muscle strength, NHISS, MRS, perceived limb heaviness, and EAQ scores. Categorical data, such as gender, educational background, hypertension, diabetes, and perceived limb heaviness, were examined by individual Chi-squared tests, and group differences between continuous variables, such as age, time from onset to enrollment, muscular strength of the limbs, NHISS, and MRS, were examined by analysis of variance (ANOVA) and nonparametric tests.

Limb muscle strength, perceived limb heaviness, and EAQ scores were examined using multiple linear regression analysis, which was used to examine the association between perceived limb heaviness and rehabilitation adherence changes with backward stepwise selection (*p* > 0.10 for exclusion).

## Results

### Clinical characteristics of participants

**Figure 1:**
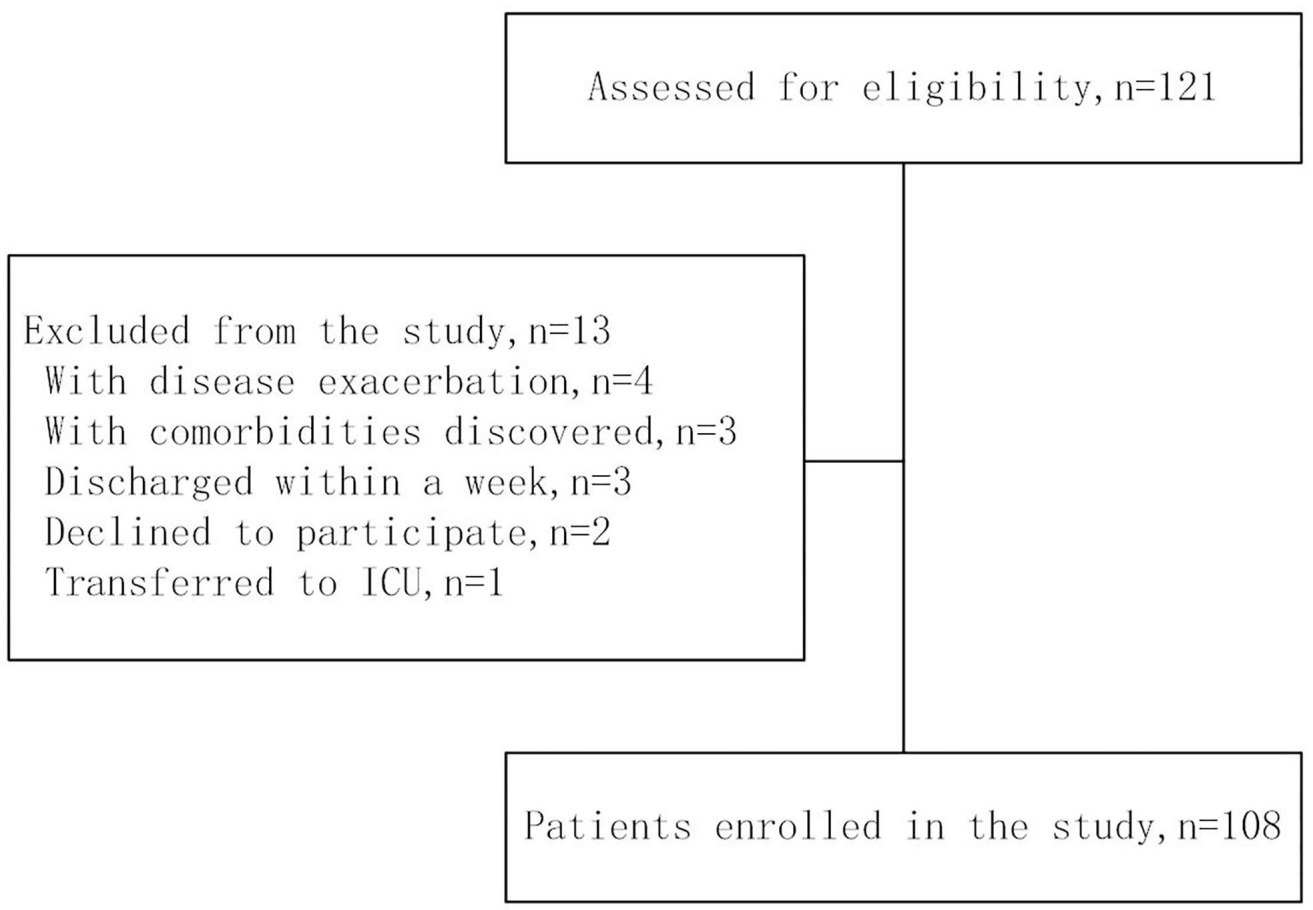
Flow chart of participants.

We administered the questionnaire to 121 participants. Four patients with stroke with disease exacerbation were excluded from the study. Three patients with stroke with comorbidities discovered after enrollment (e.g., deep venous thrombosis, Cordis mural thrombus, etc.) that could affect rehabilitation were excluded. Three patients with stroke were discharged within a week. Two patients with stroke were declined to participate. In addition, one patient was excluded because the patient was transferred to the intensive care unit. As a result, 108 subjects with stroke were enrolled in the present study (Figure 1).

Approximately 73.1% of the participants were men, with a mean age of 62.10 ± 13.3 years. The mean time from onset to enrollment was 5.22 ± 2.4 days. The mean score of the MRC scale was 3.05 ± 1.7 and 3.64 ± 1.4 for the upper limbs and the lower limbs, respectively. The mean NHISS was 6.59 ± 3.5. The mean MRS was 3.85 ± 0.4. Comorbidities were hypertension (67.6%) and diabetes (25.9%).The mean EAQ score was 34.27 ± 8.9. About 40 (37.0%) patients with stroke felt limb heaviness. Demographic characteristics of the 108 patients with stroke are summarized in Table 1.

**Table 1:** Stratification of baseline characteristics by the outcome and rehabilitation adherence level.

	**All patients (*n* = 108)**	**Low (*n* = 23)**	**Moderate (*n* = 69)**	**High (*n* = 16)**	***P*-value**
Men,%	73.1	65.2	75.4	75	0.626*
Age, years	62.10 ± 13.3	58.09 ± 15.2	64.20 ± 13.2	58.81 ± 8.1	0.405**
Time from onset to enrollment (d), mean ± SD	5.22 ± 2.4	5.35 ± 2.9	5.35 ± 2.4	4.50 ± 1.8	0.773**
muscle strength of the upper limbs, mean ± SD	3.05 ± 1.7	2.43 ± 1.9	3.06 ± 1.6	3.75 ± 1.3	0.043**^#^
muscle strength of the lower limbs, mean ± SD	3.64 ± 1.4	3.04 ± 1.6	3.78 ± 1.3	3.88 ± 1.1	0.376**
Hypertension,%	67.6	52.2	73.9	62.5	0.139*
Diabetes,%	25.9	26.1	30.0	12.5	0.399*
Education background					0.261*
Primary school and below	43	9	30	4	
Middle school	53	13	32	8	
College degree and above	12	1	7	4	
NHISS, mean ± SD	6.59 ± 3.5	7.87 ± 4.0	6.22 ± 3.1	6.38 ± 3.8	0.251***
MRS, mean ± SD	3.85 ± 0.4	3.83 ± 0.4	3.87 ± 0.4	3.81 ± 0.4	0.850***
Perceived limb heaviness					<0.001*^#^
Yes	40	18	21	1	
No	68	5	48	15	
EAQ, mean ± SD	34.27 ± 8.9	22.74 ± 3.7	34.75 ± 4.7	48.75 ± 3.9	<0.001***^#^

^*^Chi-squared tests, ^**^ ANOVAs, ^***^nonparametric tests, ^#^P <0.05.

### Analysis of the relationship between upper limb muscle strength, perceived limb heaviness, and rehabilitation adherence

**Figure 2:**
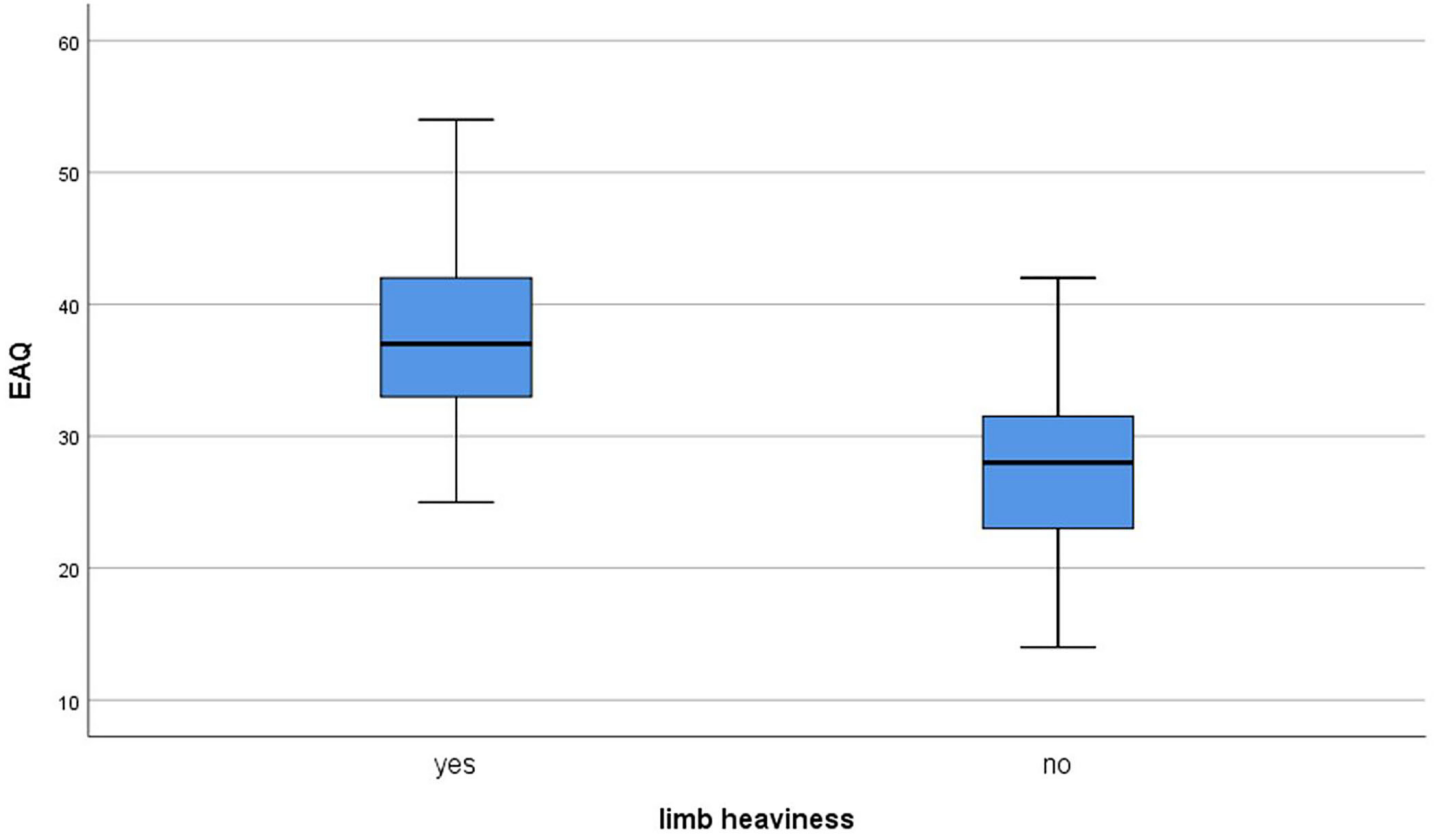
The box of the relationship between perceived limb heaviness and rehabilitation adherence.

After adjustment for these independent variables, the rehabilitation adherence score was associated with perceived limb heaviness and limb muscle strength was not associated with perceived limb heaviness (Table 2). As the obtained variance inflation factor (VIF) values were all <2, it was concluded that there was no significant multicollinearity among the independent regressors. *R*^2^ was 0.332 and 0.074 in case of the model with upper limb muscle strength and perceived limb heaviness and the model without perceived limb heaviness, respectively (Table 3). The relationship between perceived limb heaviness and rehabilitation adherence is illustrated in Figure 2.

**Table 2:** Multivariate analysis of changes in rehabilitation adherence.

	**Unstandardized**		**Standardized**		
	**coefficient**		**coefficient**		
**Factor**	**B**	**SE**		***P*-value**	**VIF**
Muscle grade of the upper limbs	0.820	0.434	0.154	0.061	1.068
Perceived limb heaviness	−9.681	1.494	−0.529	<0.001	1.068
Constant	35.355	1.706	N/A	<0.001	N/A

**Table 3:** Comparison *R*^2^ of the whole model and the model without limb heaviness.

**Model**	***R* ^2^**	**SE**	**Durbin-watson**
Whole model	0.332	7.258	2.379
Model without perceived limb heaviness	0.074	8.546	2.360

## Discussion

In this study, the main findings were as follows: (1) perceived limb heaviness, as an explanatory variable, is independently associated with a greater change in rehabilitation adherence; (2) limb muscle strength may not affect rehabilitation adherence, which means that the rehabilitation adherence level did not vary with muscle strength; and (3) demographic data and functional status may not affect the rehabilitation adherence level.

The results of this study showed us an interesting conclusion that the perception of limb heaviness as a common symptom after stroke is strongly related to rehabilitation adherence (Table 2). Limb heaviness in stroke is commonly considered as a manifestation of muscle weakness (25). However, a study by Kuppuswamy found that limb heaviness was not due to muscle weakness, perhaps a sensorimotor disorder of central origin (19). Our previous research also emphasizes that the change in muscle strength would not promote the occurrence of limb heaviness (26). In other words, patients with stroke with motor perception disorders would feel their limbs heavier. Rehabilitation after a stroke requires a lot of muscle activity and movement speed, which would cause the patient to physically slow down and feel tired (27). If patients with stroke felt limb heaviness, the sense of tiredness could be more severe. Patient feeling tired was a common barrier to vigorous exercise after a stroke and altered rehabilitation adherence (19). That is, perceived limb heaviness as a sensorimotor problem would result in less incentive for rehabilitation (28, 29). Therefore, we have reasons to believe that perceived limb heaviness alters rehabilitation adherence after stroke.

The research also found that the rehabilitation adherence level may not have a relationship with limb muscle strength. These results differed from previous studies in which muscle problems were a factor (16, 17). The most likely explanation was that patients with stroke in this study were in the acute stage. The intensity of rehabilitation in this stage was not very high (30, 31), and muscle strength was considered when selecting rehabilitation programs (22). The muscle strength of some patients with stroke would obviously improve through treatment, which would also provide positive feedback for rehabilitation adherence. Thus, the effects of muscle strength on rehabilitation adherence may not have appeared in the acute stage.

In addition, the difference in the level of rehabilitation adherence did not differ among ages (*p* = 0.405; Table 1), suggesting that the rehabilitation adherence level does not change with age. Because men and women did not differ (*p* = 0.626) and probably because most of the participants were men (73.1%), the imbalance in the men–women ratio may not result in differences in rehabilitation adherence levels. Rehabilitation adherence levels were not different in MRS (*p* = 0.850). Low levels of rehabilitation adherence would worsen their ability in ADL performance. However, ADL before rehabilitation may have little effect on adherence. About 78.7% of patients with stroke in our study had moderate and high adherence after week 1 (Table 1). There was also no difference in NHISS (*p* = 0.251). A reasonable explanation was that the effects of a neurological deficit on rehabilitation adherence were less apparent in the acute stage.

## Implications for practice

This study provides emerging evidence for perceived limb heaviness that alters rehabilitation adherence, which may influence the effects of rehabilitation. Doctors, therapists, and nurses should pay more attention to patients with stroke who perceived limb heaviness during rehabilitation. To improve their adherence to rehabilitation, effective measures to decrease the perception of heaviness should be developed.

## Limitations

This study has some limitations. First, we included only patients with stroke in the neurology department and not those with stroke in other departments or hospitals, which may bias the representation of whole hospitalized individuals and may not generalize our findings to all patients with stroke. Second, data were collected using self-report questionnaires, which may not reflect the real situation. Finally, the sample size may be bigger and the period time may be longer to get more stable results.

## Conclusion

In conclusion, perceived limb heaviness in patients with stroke leads to a low level of rehabilitation adherence. This finding may provide new information for rehabilitation management. When implementing rehabilitation, more attention should be paid to patients with stroke who perceived limb heaviness, and effective interventions should be provided to improve the level of rehabilitation adherence.

## Data availability statement

The raw data supporting the conclusions of this article will be made available by the authors, without undue reservation.

## Ethics statement

The studies involving human participants were reviewed and approved by the Human Ethics Committee of the Second Affiliated Hospital of Zhejiang University. The patients/participants provided their written informed consent to participate in this study.

## Author contributions

YuC: data curation and software. HW and YaC: investigation. ML and HY: methodology and supervision. HY and YuC: writing–original draft. ML: writing–review and editing. All authors contributed to the article and approved the submitted version.

## Funding

The research was funded by the National Natural Science Foundation of China (No: 81871839) and the medical and health science and technology planning project of the Zhejiang province (2019320070).

## Conflict of interest

The authors declare that the research was conducted in the absence of any commercial or financial relationships that could be construed as a potential conflict of interest.

## Publisher's note

All claims expressed in this article are solely those of the authors and do not necessarily represent those of their affiliated organizations, or those of the publisher, the editors and the reviewers. Any product that may be evaluated in this article, or claim that may be made by its manufacturer, is not guaranteed or endorsed by the publisher.
